# Challenging Differential Diagnosis of Mandible Angle Metastasis from Breast Cancer

**DOI:** 10.1155/2024/2667323

**Published:** 2024-01-25

**Authors:** Francesca Spirito, Mariateresa Ambrosino, Federica Morrone, Roberto Duraccio, Lorenzo Lo Muzio, Antonio Della Valle

**Affiliations:** ^1^Department of Clinical and Experimental Medicine, University of Foggia, Via Rovelli, Foggia 71122, Italy; ^2^Unit of Radiology, University of Naples Federico II, Naples, Italy

## Abstract

Breast cancer is the most common cancer in women and the second leading cause of cancer-related death. Breast cancer manifestations in the head and neck are relatively rare, and they are mostly bony metastasis to the mandible and maxilla. In this paper, we present a case report of a metastatic tumor in the mandibular angle originating from breast carcinoma. A 32-year-old female patient with a paresthesia/anesthesia in the left mandibular area was referred to us to aid in the differential diagnosis between osteonecrosis and metastasis. Her medical history revealed a radical bimastectomy 3 years ago for invasive lobular carcinoma of the breasts. Additionally, she received chemotherapy and radiotherapy 3 years ago, and intravenous zoledronic acid was administered every 3 weeks. Intraoral examination did not reveal any mucosal ulcer or fistula, and there was no radiological evidence of cyst. The patient demonstrated good oral hygiene. Palpable regional left submandibular lymph nodes and a few swellings on the lateral angular mandibular surface were observed. Cone-beam computed tomography (CBCT) and positron emission tomography (PET) were performed. CBCT showed small poorly diffused radiopacity in proximity to the mandibular angle on both medial and lateral surfaces. PET showed fluoro-2-deoxy-D-glucose uptake in the mandible in the left angle surface area. Based on the patient's clinical history, signs, symptoms, and tomographic evidence, we were able to diagnose mandibular metastasis. This case also highlights the importance of proficiency in reading tomographic examinations, which can be carried out in dental clinics for various purposes. In the absence of symptoms, misdiagnosis can occur, underscoring the significance of accurate interpretation and diagnosis.

## 1. Introduction

Metastasis to oral and maxillofacial region is a rare finding, representing approximately 1/1.5% of all malignancies in the maxillofacial region [1, 2]. Among the oral sites, the mandible is the most commonly affected [3], accounting for 44.9% of metastatic lesions in the oromaxillofacial region [4], followed by the maxilla (24%) [5] and gingival soft tissue (16.8%) [4]. Due to its rarity, the diagnosis of metastatic lesions is challenging for both the clinician and the pathologist. The most common primary sites of malignancy origin are the lung, representing 17.3% of the cases, followed by the breast (12.6%), the kidney (12.5%), and the liver (8.9%) [6]. Breast cancer is the most prevalent cancer in women and the second leading cause of cancer-related death. In 2020, 2,261,419 women were diagnosed with breast cancer all around the word [7]. At the time of diagnosis, 4-6% of women with breast cancer have distant metastases, with many developing distant disease later on [8]. Metastasis in breast cancer is commonly seen in the lungs, liver, bones, pleura, brain, and kidneys and is rarely seen in adrenal glands and ovaries [8]. Distant metastases are relatively common in breast cancer, but spread to the head and neck region is uncommon, in which supraclavicular lymphadenopathy and bony metastases to the mandible and maxilla are the most common manifestation [9]. The mandible seems affected more frequently than the maxilla, with the predilection for the regions such as distal, angle, and ramus, due to factors like the deposition of cancerous cells causing the presence of hematopoietic bone marrow, anatomical subdivision of local blood vessels, and reduced velocity of blood flow [10, 11]. The diagnosis of mandibular metastasis is challenging due to its nonspecific clinical symptomatology, and it is characterized by a high clinical latency causing a delay in diagnosis. Clinical signs and symptoms associated with metastatic tumors of the maxillary bones include swelling, orofacial pain, altered neurosensorial, tumors of soft tissues, ulceration, regional lymphadenopathy, trismus, and, in some cases, pathological fractures [10, 12, 13]. Numbness, paresthesia, and anesthesia of the lower lip and chin are considered important and pathognomonic signs of metastatic disease [10, 12, 13]. It is worth noting that metastatic breast carcinoma lesions can sometimes mimic inflammatory or infectious diseases of the jaws [14]. Additionally, the disease might be totally asymptomatic [10]. Breast carcinoma can stimulate bone formation in distance metastasis [15], resulting in a varied radiographic appearance in the jaws, ranging from well circumscribed to poorly circumscribed radiolucencies or poorly diffused radiopacity, the latter being referred to as a moth-eaten radioappearance [16]. It is also possible that metastasis not to be visible on radiographs [1].

The identification of mandible metastasis poses a challenge for clinicians, given the absence of precise clinical and radiological indicators. This difficulty in diagnosis can result in delays in treatment, ultimately exacerbating the prognosis of the disease. Consequently, pinpointing the primary tumor becomes arduous due to this atypical presentation.

In this paper, we aim to present a case of metastatic tumor in the mandibular angle originating from breast carcinoma.

## 2. Case Presentation

**Figure 1:**
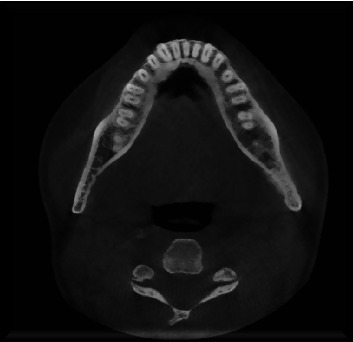
Noticeable and substantial thickening of the bony trabeculation observed at the left mandibular angle.

**Figure 2:**
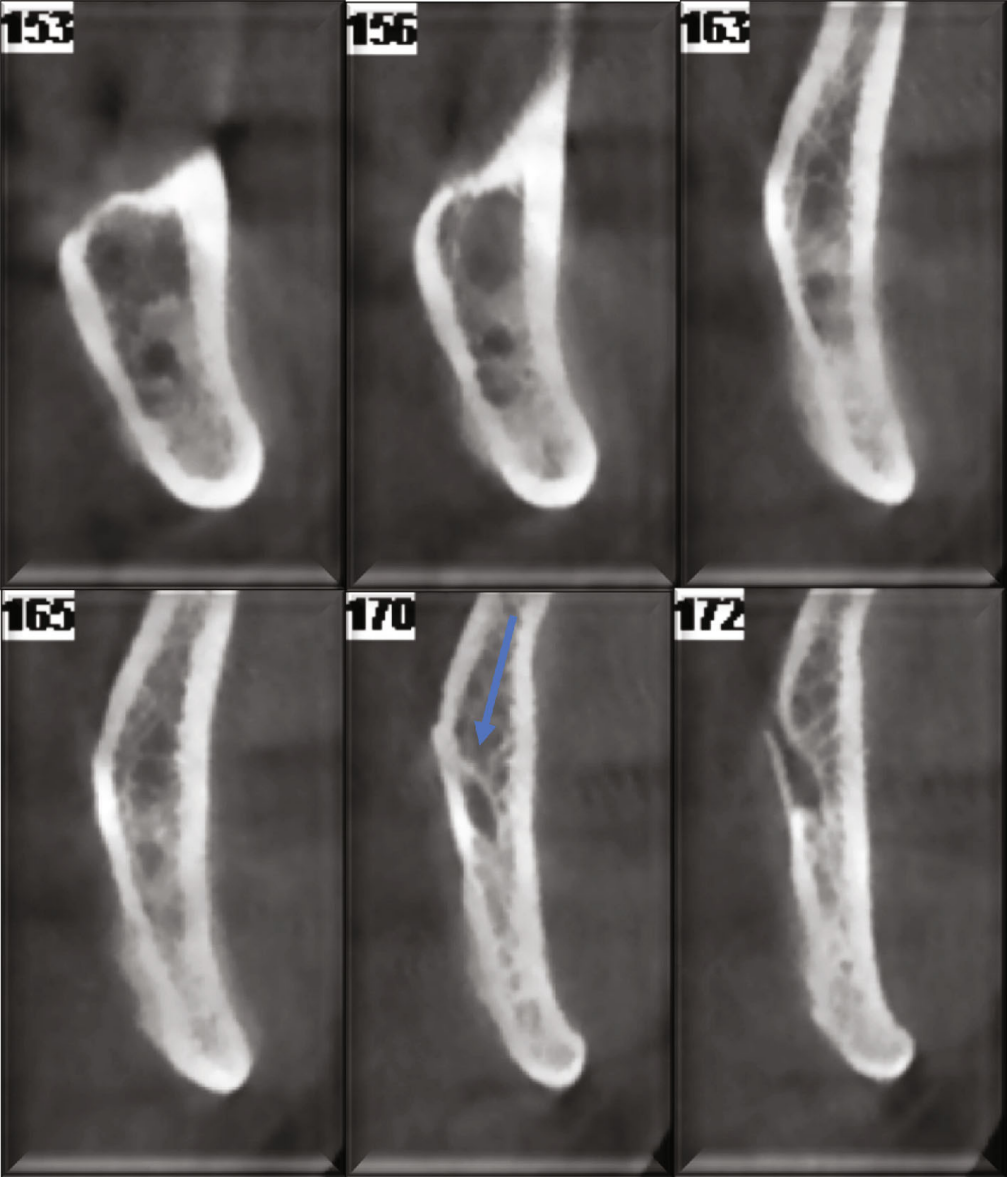
Parasagittal sections reveal an irregular reduction in the width of the mandibular canal at the level of the mandibular angle, extending approximately 3 mm with the apparent engagement of the canal. Additionally, there is a noticeable periosteal reaction, more evident on the lingual side, as well as on the vestibular side. Furthermore, the mandibular cortex displays an irregular appearance with frayed edges, particularly prominent on the lingual side.

**Figure 3:**
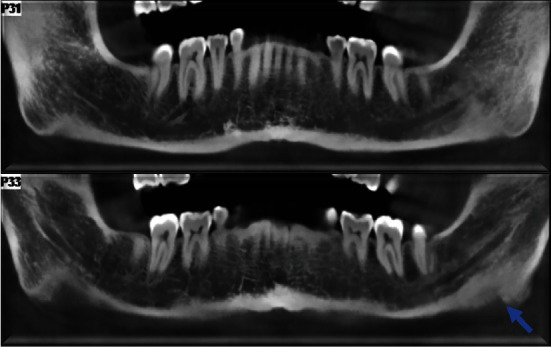
The thickened bony trabeculation exhibits an anterior extension at the mandibular pericanal level.

**Figure 4:**
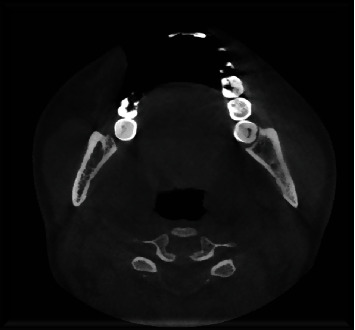
The alteration in trabecular osteoabsorption is evident in a cranial direction, extending up to approximately 5 mm from the Spix plug in a diffuse manner.

A 32-year-old female patient presented with paresthesia/anesthesia in the left mandibular area for the past five days when she was referred to our oral surgery and pathology office by her oncologist to allow the differential diagnosis of osteonecrosis or metastasis. Her medical history revealed a radical bimastectomy 3 years ago for invasive lobular carcinoma of the breasts. The tumor was negative for the expression of estrogen, progesterone, and human epidermal growth factor receptor 2 (c-erb B2) (HER2/neu) in immunohistochemistry. She had received chemotherapy and radiotherapy three years ago, and chemotherapy was restarted after only one year. Additionally, she received 4 mg of intravenous zoledronic acid every three weeks for bone metastasis prevention. An inspection of the oral cavity did not reveal any mucosal ulcers, fistulas, cysts, periapical, and/or periodontal infectious disease, or swelling from tumors, and her oral hygiene was good. Palpable regional left submandibular lymph nodes and a few swellings on the lateral angular mandibular surface were detected. Based on the patient's medical history, paresthesia of the lower lip and chin, and the radiographic appearance and location of the lesion, metastatic disease was highly suspected. To confirm the diagnosis suspect, cone-beam computed tomography (CBCT) and positron emission tomography (PET) were employed, both of which showed positive results. No lytic lesions of the mandible were observed. Sagittal 1 mm thick CBCT showed small, poorly diffused radiopacity near the mandibular angle on both medial and lateral surfaces (Figures 1–4). PET results showed fluoro-2-deoxy-D-glucose uptake in the left angle surface area of the mandible. In the present case, history of breast cancer was known, and therefore, a fine needle aspiration cytology (FNAC) was performed. Additionally, the patient exhibited radiological signs of other heteroplasias for which also a FNAC was conducted, yielding a positive result for breast carcinoma features. The FNAC findings showed clusters of loosely cohesive tumor cells displaying moderate pleomorphism and prominent nucleoli. Positive pancytokeratin staining in the tumor cells was indicative of metastatic carcinoma. Based on the clinical history, radiological appearance, and cytological analysis of the lesions, a diagnosis of metastatic lesion to the mandible was established. Surgical management was not deemed appropriate, and the patient was referred to her oncologist for further treatment.

## 3. Discussion

Jaw metastatic lesions often present vague or meaningless symptoms, which can mimic dental infections [10, 12–14]. Metastases to the oral cavity and the jaws account for approximately 1% of all diagnosed oral malignancies [3]. The mandible is more commonly affected than the upper jaw, with a higher tendency in the posterior areas [15]. The posterior areas are more susceptible to the evolution of cancerous cells due to the presence of hematopoietic bone marrow, which exhibits reduced blood flow velocity [10]. Breast cancer metastasizes to the mandibles three times as often as any other malignant tumor [3]. Metastatic jaw tumors typically present with pain, numbness or paresthesia of the lower lip, trismus, irritation, ulceration, exophytic growth, and halitosis [10, 12–14]. In our case, the female patient only complained of oral-facial pain in the left temporomandibular area, along with numbness and paresthesia/anesthesia of the ipsilateral lower lip. The CBCT exam revealed a “moth-eaten” appearance in the mandibular bone angle. Metastatic carcinomas from the breast can stimulate bone formation resulting in mixed radiopaque/radiolucent lesions [16]. A comprehensive medical history, along with proper clinical and laboratory investigations, including immunohistochemical staining, can aid in the diagnosis. Most patients with a metastatic tumor in the oral cavity also develop metastases at other sites, often leaving palliation as the only option [17]. Regardless of the site of origin, oral cavity metastases are reported as the first clinical symptom of a primary malignancy in 22-33% of patients [11]; several authors have reported that adenocarcinoma is the most common histologic type and that breast adenocarcinoma is the most common malignancy that metastasizes to the mandible or maxilla [11]. The primary focus should be on pain relief and the prevention of possible infections, fractures, or hemorrhage. The diagnosis of metastasis to the mandibular bone is difficult in the absence of clinical symptoms and of clinical characteristics as pain, swelling, paresthesia, foul smell, gingival irritation, tooth mobility, exophytic growths of the soft tissues, reduced mouth opening, and infrequently pathological fractures [8, 11]. In this specific case, the paresthesia of the left lower lip and chin were the only signs that prompted us to carry out an in-depth instrumental radiological exam. The radiographic appearance of the metastatic disease in the mandibular angle can be well circumscribed or poorly circumscribed radiolucencies, and it is often referred to as “moth-eaten” appearance [16]. Oncologists seek advice in oral surgery for the evaluation of possible osteonecrosis of the jaw caused by bisphosphonate treatment. The existence of exposed necrotic bone over 8 weeks with past or recent use of bisphosphonates is an essential element for the diagnosis of medication-related osteonecrosis of the jaw. In cases where the patient does not present obvious symptoms and the tomographic examination is carried out for other purposes, it becomes challenging for an oral surgeon to make a diagnosis, resulting in diagnostic delay. In conclusion, this case highlights the importance of thoroughly and carefully analyzing instrumental data, considering the patient's clinical, surgical, oncopathological, and oncotherapeutic history. Without symptoms such as dysesthesia of the lower lip or pain in the regional area of the mandibular angle, there is a risk of diagnostic omission and delayed detection of pathological findings. In dental surgeries, patients with a clinical history of cancer should receive high attention in terms of instrumental data throughout the diagnostic and therapeutic pathway. Omitting such evidence, even if not marked by clinical pathology, exposes the practitioner to a high degree of professional liability.

## Abbreviations

CBCT: Cone-beam computed tomography
PET: Positron emission tomography.
